# A comparative study on antioxidant properties, total phenolics, total flavonoid contents, and cytotoxic properties of marine green microalgae and diatoms

**DOI:** 10.1016/j.jgeb.2024.100456

**Published:** 2025-01-22

**Authors:** Umme Tamanna Ferdous, Armania Nurdin, Saila Ismail, Khozirah Shaari, Zetty Norhana Balia Yusof

**Affiliations:** a Center for Biosystems and Machines (IRC-BSM), King Fahd University of Petroleum & Minerals, Dhahran, Saudi Arabia; b Department of Biomedical Sciences, Faculty of Medicine and Health Sciences, Universiti Putra Malaysia, 43400 UPM Serdang, Selangor, Malaysia; c Laboratory of UPM-MAKNA Cancer Research (CANRES), Institute of Bioscience, Universiti Putra Malaysia, 43400 UPM Serdang, Selangor, Malaysia; d Department of Biochemistry, Faculty of Biotechnology and Biomolecular Sciences, Universiti Putra Malaysia, 43400 Serdang, Selangor, Malaysia; e Natural Medicines and Products Research Laboratory, Institute of Bioscience, Universiti Putra Malaysia, 43400 Serdang, Selangor, Malaysia; f Department of Chemistry, Faculty of Science, Universiti Putra Malaysia, 43400 Serdang, Selangor, Malaysia; g Aquatic Animal Health and Therapeutics Laboratory (AquaHealth), Institute of Bioscience, Universiti Putra Malaysia, 43400 Serdang, Selangor, Malaysia; h Bioprocessing and Biomanufacturing Research Complex, Universiti Putra Malaysia, 43400 Serdang, Selangor, Malaysia

**Keywords:** Antioxidant, Cytotoxicity, Diatom, Green Microalgae, Marine

## Abstract

Despite having valuable and novel metabolites, the marine microalgae species are still not thoroughly investigated for their pharmaceutical and nutraceutical importance. Therefore, this study was focused on investigating the crude extracts of marine green microalgae species, *Tetraselmis* sp., *Nannochloropsis* sp., and diatoms *Chaetoceros* sp., and *Thalassiosira* sp., isolated from the Malaysian coastal region in terms of their antioxidant activity, total phenolics, total flavonoid contents and cytotoxicity against human breast cancer cells, MCF-7. Among twenty-eight crude extracts, *Tetraselmis* ethanol and ethyl acetate extract showed the highest amount of total phenolic (19.87 mg GAE/g), and total flavonoid content (38.58 mg QE/g of extract), respectively. From the antioxidant assays, methanol and ethyl acetate extract of *Tetraselmis* sp. exhibited significantly higher (p < 0.05) antioxidant activities, revealed through DPPH (54.41 ± 1.18 mg Trolox Equivalent Antioxidant Capacity or TEAC/g extract) and ABTS (41.57 ± 0.83 mg TEAC/g extract) radical scavenging activities, respectively than the rest. Ethyl acetate extract of *Tetraselmis* sp. also showed high ferric reducing power (113.46 ± 4.83 mg TEAC/g extract). On the contrary, methanol and ethyl acetate extract of *Chaetoceros* sp. showed the highest cytotoxicity towards MCF-7 and reduced the cell viability to 21.26 % and 21.56 %, respectively. The data suggest that marine diatom *Chaetoceros* sp. has a good cytotoxic effect on MCF-7, while marine green microalga *Tetraselmis* sp. has good radical scavenging and ferric reduction capabilities, warranting further investigation along with their metabolic profiling, cancer cell killing mechanism and extensive *in vivo* study.

## Highlights

• Marine diatom *Chaetoceros* sp. showed the highest cytotoxicity against breast cancer cells.
• Green microalgae showed stronger antioxidant activity than diatom.
• Medium polar solvents like ethanol, methanol, acetone, and ethyl acetate extracted more phytochemicals.
• The cytotoxic effect of *Thalassiosira* sp. extracts was investigated and ethanol extract showed good cytotoxicity.

## 1 Introduction

Marine organisms reside in a salty aqueous environment that covers 71 % of the earth’s surface and accounts for 90 % of the earth’s biosphere^1^. This is a gigantic reservoir for diversified marine species, with approximately 2500,000 species so far.^2^ Marine microalgae account for a significant portion of oceanic biomass. Microalgae, both eukaryotic and cyanobacteria, comprise more than 30,000 species and contribute up to 40 % of global productivity.^3^ Additionally, they can withstand all environmental extremities, from cold to hydrothermal vents. On a lab-scale or industrial scale, they can be grown all year round irrespective of any seasonal variation, which also excludes the need for long-term storage and helps to avoid valuable phytochemical degradation. They can be grown with a limited nutritional supply and the advantageous point is that microalgae can be grown in wastewater as a nutrient source, which in turn, reduces carbon footprint and water usage.^4^ Not only that, microalgae can be grown in large photo bioreactors without competing with arable land and disturbing the human food chain. Moreover, microalgae can grow faster than terrestrial plants.^5^.

The oceanic ecosystem is characterized as a hostile and unpleasant place where the marine flora responds to the constant presence of predators, high pH, water pressure, shortage of sunlight, and nutrient deficiency by developing symbiotic and adaptive mechanisms. Their defense mechanism to survive in this environment aids in the production of a wide variety of secondary metabolites.^6^ These secondary metabolites from marine organisms are now exploited to design life-saving drugs and drug leads. Marine microalgae contain a wide range of phytochemicals like carotenoids, phenolics, flavonoids, fatty acids, alkaloids, polysaccharides, and vitamins. These phytochemicals make them attractive sources of bioactive compounds that are frequently used in the cosmetic, aquaculture, and energy-related industry.^7^, ^8^ They can produce pharmaceutically important phytochemicals, especially anticancer compounds.^9^ Hamidi et al., (2020) mentioned that marine microalgae may produce more carotenoids and EPA than marine bacteria.^10^ Eukaryotic microalgae have also been known for their low toxicity. For instance, *Chlorella* sp. is considered as generally recognized as safe (GRAS) which is approved by the U.S. Food and Drug Administration (FDA). No toxin is found from the microalgae species like, *Isochrysis* sp., *Nannochloropsis* sp., *Tetraselmis* sp., and *Thalassiosira* sp.. These microalgae including *Chaetoceros* sp. are now frequently used in aquaculture industries as fish feed.^11^ Due to the presence of antioxidants, microalgal biomass is used popularly as dietary supplements and also as food additives.^7^ Green eukaryotic microalgae, *Chlorella* sp., *Nannochloropsis* sp., *Tetraselmis* sp. are now used as commercial food supplements, while brown microalgae *Isochrysis* sp. is used as food additive.^12^, ^13^ Fortification of food products, like bread, cookies, pasta, snacks, yogurt, and ice cream with microalgal antioxidants augments nutritional status and sensorial quality.^14^ Microalga-derived antioxidants are now also used in preparing cosmetic formulation. *Chlorella* sp., *Spirulina* sp., *Nannochloropsis* sp., and *Chlamydomonas nivalis* are now being commercialized and popularly used as cosmetics ingredients due to their moisturizing, anti-aging and UV-protective properties^15^. Antioxidant supplementation following cancer therapy can improve patient outcomes and survival rates by lessening oxidative damage to adjacent healthy tissues and minimizing negative effects. According to certain research, these supplements can cause tumor cells to undergo apoptosis, restrict cell development, and suppress cell proliferation.^16^.

Indigenous eukaryotic marine microalgae species, *Isochrysis galbana* and *Chaetoceros calcitrans,* isolated from Malaysian coastal areas have shown good fatty acids profile^17^, ^18^, ^19^ Total phenolic content and high antioxidant activities were reported for indigenous marine *Tetraselmis tetrathele*, *Nannochloropsis* sp. and *Chaetoceros calcitrans.*^20^, ^21^, ^22^ *Tetraselmis* species are important due to their higher protein, lipids, essential fatty acids, sterol and a high content of carotenoids such as the xanthophylls lutein, violaxanthin, neoxanthin, antheraxanthin and loroxanthin esters, which show strong antioxidant activity.^23^ *N. oculata* and *N. gaditana* are now commercially produced to be sold as a health supplements because these species are rich in omega-3 fatty acid and EPA, which are known to have many health benefits.^13^ *Chaetoceros* and *Thalassiosira*, two diatoms, are commonly cultivated as live feed for bivalves and crustaceans due to its higher content of polyunsaturated fatty acids.^24^, ^25^ Diatom is a group of unicellular eukaryotic microalgae which has distinct silica cell walls.^26^ *Chaetoceros calcitrans* is the most studied species and its antioxidant and cytotoxic activities were documented in previous studies.^21^ There are some reports of good antioxidant activity of *Thalassiosira* sp., but study on its anticancer activity are scarce.^27^ Hossain et al., (2020) also highlighted the suitability of Malaysian weather and location for microalgal growth in terms of nutrient availability, solar irradiance, salinity, and temperature.^28^ However, these microalgae remain unexplored vastly in terms of their bioactivities. More research on eukaryotic, especially edible microalgae species and their bioactivities is warranted. Therefore, this study aims to investigate and compare the antioxidant and cytotoxic activities of the crude extracts from marine indigenous eukaryotic green microalgae, *Tetraselmis* sp., *Nannochloropsis* sp., and diatoms *Chaetoceros* sp*.,* and *Thalassiosira* sp*.* as well as their total phenolic and total flavonoid contents.

## 2 Methods

### 2.1 Microalgae Culture condition

Indigenous isolates of the marine green microalga species *Tetraselmis* sp., *Nannochloropsis* sp., and *Thalassiosira* sp. were obtained from the International Institute of Aquaculture (I-AQUAS) and Aquatic Science of Universiti Putra Malaysia, Teluk Kemang, Port Dickson. *Chaetoceros* sp. was collected from Aquatic Animal Health (AAHU), Faculty of Veterinary Medicine, Universiti Putra Malaysia (Table 1). The microalgae species were grown first in 250 ml Erlenmeyer flasks and gradually scaled up to 1000 ml Erlenmeyer flasks with fresh growth media, and grown under the following culture condition for two weeks with continuous shaking in an orbital shaker. *Tetraselmis* sp. and *Nannochloropsis* sp. were grown in F/2 media. For the diatoms, silica was added in F/2 media (Supplementary Table 1).

**Table 1:** Culture Condition of the selected marine microalgae.

Microalgae	Source	Culture Media	Culture condition
Green Microalgae			
*Tetraselmis* sp.	I-AQUAS, Port Dickson	F/2	24 μmol photons/ m^2^/ s, 24 ± 2 °C, 130 rpm
*Nannochloropsis* sp.	I-AQUAS, Port Dickson	F/2	24 μmol photons/ m^2^/ s, 24 ± 2 °C, 130 rpm
Diatoms			
*Chaetoceros* sp.	Aquatic Animal Health (AAHU), Faculty of Veterinary Medicine, Universiti Putra Malaysia	F/2 with silica	20 μmol photons/ m^2^/ s, 24 ± 2 °C, 130 rpm
*Thalassiosira* sp.	I-AQUAS, Port Dickson	F/2 with silica	20 μmol photons/ m^2^/ s, 24 ± 2 °C, 130 rpm

### 2.2 Morphological characterization

The microalgal species' morphological characterization, such as cell shape, size, motility, and appendages, was carried out by examining the cells at a 100x magnification using a Carl Zeiss bright field microscope (Oberkochen, Germany).

### 2.3 Crude extract Preparation

Seven distinct solvents with varying polarity were used to prepare the crude extracts of the microalgae species: methanol, ethanol, acetone, hexane, dichloromethane (DCM), chloroform, and ethyl acetate, following the previously described protocol. In short, microalgal biomass was harvested and 10 g of freeze-dried biomass was ground. One hundred milligrams of microalgal powder were added to ten milliliters of each solvent (100 %). The mixture was sonicated in an ultrasonic water bath (Thermo Fisher, USA) for 20 min in cold conditions and later shaken for an hour at room temperature in a shaker. The extract-containing supernatant was then separated after the extract mixture was centrifuged at 1209 g for ten minutes at 4 °C (Centurion, UK). The remaining pellet was extracted again two times. Following each extraction, the supernatants were combined and filtered using Whatman filter No. 1 paper. The extracts were dried using a BÜCHI rotary evaporator (Switzerland). Weighing each crude extract, it was stored at −20 °C for further examination. The yield of extracts was determined as follows:

$$\mathrm{Extractionyield}\left(\%\right)=\frac{\mathrm{weightoffreezedriedextract}}{\mathrm{weightofdriedbiomass}}\times 100$$

### 2.4 Quantification of TPC and TFC

Several gallic acid and quercetin concentrations were used as standard at 200, 100, 50, 25, 12.5, 6.25 and 3.125 μg/ml. To measure TPC content, forty microliters of 10 % (v/v) FC reagent were added to each 20 µl sample, together with 160 µl of NaHCO_3_ (700 mM) solution, before incubation at room temperature for two hours in the dark. The OD reading at 765 nm was acquired by Multiskan™ GO plate reader (Thermo Fisher Scientific, Finland) and expressed as the mg GAE/ g of extract. Gallic acid (200–3.125 μg/ml) was used as a standard. To measure TFC content, algal extract 20 µl, AlCl_3_ −10 % (20 µL), distilled water (180 µL), and Sodium acetate (1 M) were mixed and incubated at 30 mins at room temperature. The OD reading at 415 nm and expressed as the mg QE/ g of extract.^29^.

### 2.5 Antioxidant assays

The ability of microalgae extracts to scavenge DPPH and ABTS radicals, and to reduce ferric ions was measured in accordance with previous reports. Trolox served as standard (250–3.9 μg/ml) and the data were presented as mg TEAC/g of extract. A DPPH solution (0.1 mM) was quickly produced in methanol and utilized immediately for DPPH assay. To 195 µl of the produced DPPH, 50 Âµl of microalgal extract (500 μg/ml) were mixed. The OD was obtained at 540 nm after an hour of incubation at 25 °C in the dark. The inhibition % of DPPH was calculated using the following formula.

$$Inhibition\left(\%\right)=\frac{\mathrm{Acontrol}-Atest}{\mathrm{Acontrol}}\times 100$$

here, A_Control_ = OD of DPPH; A_Test_ = OD of DPPH and the extract

To measure Fe^3+^ reducing capacity, a 3:3:1:1 mixture of 1 % [K_3_Fe(CN)_6_] (w/v), 1 M HCl (v/v), 1 % SDS solution (w/v), and 0.2 % FeCl_3_ solution (w/v) was used to produce the FRAP reagent. Subsequently, 200 μL of the freshly made FRAP reagent was mixed with twenty microlitres of each extract, and the mixture was incubated at 50 °C. After 20 mins, the absorbance was measured at 750 nm.

ABTS + solution was prepared to perform ABTS assay by combining potassium persulfate (2.45 mM) with ABTS solution (7 mM) (1:1, v/v). Following a 16-hour dark incubation period, the ABTS solution's optical density was calibrated to 0.700 ± 0.005 at 734 nm. Next, 20 µL of each microalgae extract (500 μg/ml) was mixed with 200 µL of this produced ABTS solution, and the mixture was incubated for 6 min in the dark. The absorbance was taken at 734 nm.

The scavenging capacity (%) was measured using the following equation:

$$ABTS\;radical\;scavenging\;activity\left(\%\right)=\frac{\mathrm{Acontrol}-Atest}{\mathrm{Acontrol}}\times 100$$

### 2.6 Cytotoxicity assay

The human breast cancer cells, MCF-7 were seeded in a 96-well plate with a confluency of 10^4^ cells/well and incubated 24 h in a CO_2_ incubator at 37 °C. following incubation, the media was discarded and 100 μg/ml of the algal extracts containing media was added to each well and was incubated once more for 24, 48, and 72 h. 5 mg MTT powder was mixed with 1 ml of PBS to make MTT solution and 10 µl of this solution was added to each well. After incubation for 3 h without light, media was discarded carefully and DMSO was added. The OD was taken with a iMARK^TM^ plate reader (BIO-RAD) at 570 nm.^29^ The following formula was utilized to determine the cell viability:

$$Cellviability\left(\%\right)=\frac{\mathrm{ODoftreatment}}{\mathrm{ODofcontrol}}\times 100$$

### 2.7 Statistical analysis

Data obtained from at least three independent assays were computed and presented in form of mean ± SEM. Significant differences at *p* < 0.05 level were also determined using IBM SPSS v22 (USA) software by One-way ANOVA with Tukey or Dunnett *posthoc* test. Pearson correlation test was carried out to determine the correlation between antioxidant assays, DPPH, ABTS and FRAP assays, and TPC/TFC.

## 3 Results

### 3.1 Morphological identification

Based on taxonomic keys from AlgaeBase (https://www.algaebase.org) and Diatoms of North America (https://www.diatoms.org), morphological identification of the studied microalgae was carried out. Under the microscope, *Tetraselmis* sp. was found as unicellular, compressed shaped green microalga with flagella and distinct groove. The size of the microalga ranged from 12-15 μm. *Nannochloropsis* sp. was unicellular and spherical but light green than *Tetraselmi*s sp.. The size of the cells was also smaller, about 2–5 µm. *Chaetoceros* sp. was also brown in color and cylindrical in shape, and size ranging from 3-8 μm. The distinguishing characteristics of this microalgae were its setae, which had thick and long appendages from each corner of the cells. On the other hand, *Thalassiosira* sp. was found as single, short barrel-shaped brown cells with slightly round edges in this study. The cells had distinctive frustules and chloroplasts. The distinctive elliptical chloroplasts were located near the periphery. The cells were 10–15 µm. (Supplementary Fig. 1).

### 3.2 Extraction yield, TPC and TFC

The result of extraction yield from the organic solvents showed that the highest amount of extract was found in the ethanol extract of *Nannochloropsis* sp. (33 %) and the lowest amount was found in the hexane extract of the same species (5 %) and *Thalassiosira* sp. (6 %) (Table 2). Among these solvents, extraction with hexane yielded the lowest amount of extracts (ranging from 5-15 %), whereas methanol and ethanol showed the highest amount of extracts, ranging from 19-28 % and 16–33 %, respectively.

**Table 2:** Total phenolic, Total flavonoid contents and extraction yield of green microalgae and diatoms.

		Green microalgae		Diatoms	
Extracts		***Tetraselmis* sp.**	***Nannochloropsis* sp.**	***Chaetoceros* sp.**	***Thalassiosira* sp.**
Methanol	**TPC**	12.36 ± 0.55^bc^	5.05 ± 0.56^bc^	3.57 ± 0.11^d^	3.07 ± 0.32^bc^
	**TFC**	19.74 ± 0.61^cd^	15.18 ± 0.92^c^	3.93 ± 0.44^d^	6.75 ± 0.66^b^
	**Yield (%)**	21	19	28	24
Ethanol	**TPC**	19.87 ± 1.50^a^	10.04 ± 0.79^a^	5.86 ± 0.26^bc^	2.98 ± 0.2^bc^
	**TFC**	37.49 ± 1.53^a^	31.48 ± 0.86^a^	17.82 ± 1.16^b^	8.42 ± 0.78^b^
	**Yield (%)**	17	33	21	16
Acetone	**TPC**	10.13 ± 0.55^cd^	6.82 ± 0.16^b^	4.81 ± 0.55^cd^	2.04 ± 0.28^c^
	**TFC**	15.71 ± 1.13^d^	23.28 ± 0.95^b^	9.98 ± 0.66^c^	8.20 ± 1.35^b^
	**Yield (%)**	28	11	23	9
Chloroform	**TPC**	9.27 ± 0.67^cd^	6.72 ± 0.43^b^	3.27 ± 0.15^d^	4.12 ± 0.29^ab^
	**TFC**	31.37 ± 0.76^b^	29.84 ± 1.43^a^	5.41 ± 0.28^cd^	6.61 ± 0.97^b^
	**Yield (%)**	13	16	22	18
Ethyl acetate	**TPC**	15.20 ± 0.96^ab^	11.54 ± 0.55^a^	15.88 ± 0.41^a^	4.80 ± 0.45^a^
	**TFC**	38.58 ± 1.37^a^	35.04 ± 0.95^a^	34.52 ± 2.56^a^	18.76 ± 1.23^a^
	**Yield (%)**	18	16	10	18
DCM	**TPC**	7.38 ± 0.65^d^	5.72 ± 0.20^bc^	7.04 ± 0.73^b^	2.69 ± 0.33^bc^
	**TFC**	23.08 ± 0.87^c^	21.59 ± 1.86^b^	16.20 ± 0.60^b^	7.40 ± 0.49^b^
	**Yield (%)**	21	17	8	9
Hexane	**TPC**	5.67 ± 0.47^d^	3.43 ± 0.40^c^	3.44 ± 0.31^d^	3.11 ± 0.26^bc^
	**TFC**	21.00 ± 1.16^cd^	1.53 ± 0.21^d^	1.42 ± 0.24^d^	0.52 ± 0.05^c^

*TPC and TFC was expressed as mg GAE/g of extract and mg QE/g of extract, respectively. The significant difference among the crude extracts’ TPC and TFC values in the same column is specified by the superscripts ^(a,b,c,d,e,f)^ (p < 0.05).

Total extractable phenolics in the marine microalgae species were assessed using the linear standard curve of gallic acid, (y = 0.0073x + 0.005, R^2^ = 0.9999). TPC of all studied extracts ranged from 2.04 to 19.87 mg GAE/g of extract. TPC detected in ethanol extract of *Tetraselmis* sp. (19.87 mg GAE/g) was the highest in amount, followed by ethyl acetate extract of *Chaetoceros* sp. (15.88 mg GAE/g of extract) and *Tetraselmis* sp. (15.2 mg GAE/g of extract). The lowest was found in the acetone extract of *Thalassiosira* sp. (2.04 mg GAE/g of extract). It is observed that polar solvents, like ethanol, methanol and ethyl acetate extracted more phenolics than non-polar solvents like hexane and dichloromethane.

In this study, total flavonoid contents in the selected marine microalgae species were determined using a linear standard curve of quercetin (y = 0.0034x + 0.0125, R^2^ = 0.9973). Like TPC, *Tetraselmis* sp. ethyl acetate extract showed high TFC contents (38.58 mg QE/g of extract), followed by the ethanol extract of *Tetraselmis* sp. (37.49 mg QE/g of extract) and ethyl acetate of *Nannochloropsis* sp. (35.04 mg QE/g of extract). The lowest TFC was found in the hexane extract of *Thalassiosira* sp. (0.52 mg QE/g of extract) (Table 2).

### 3.3 Antioxidant activity of marine green microalgae and diatom crude extracts

The DPPH radical scavenging activity in the marine microalgae species was assessed using the linear standard curve of Trolox, (y = 1.4116x + 2.4976; R^2^ = 0.9901). The methanolic extract was found the best DPPH scavenger for all the species (Table 3). *Tetraselmis* sp. methanolic extract exhibited the highest DPPH scavenging capacity (54.41 mg TEAC/ g of Extract), followed by methanolic extract of *Nannochloropsis* sp. (46.28 mg TEAC/g of Extract). Hexane extracts of both green microalgae and diatoms showed lower DPPH scavenging activity. The lowest activity was observed in the chloroform extract of *Thalassiosira* sp. (0.83 mg TEAC/g of Extract) while no activity was observed in the hexane extract.

**Table 3:** Antioxidant activity of marine green microalgae and diatom extracts of different polarity solvents *in vitro.*

Extracts		*Tetraselmis* sp.	*Nannochloropsis* sp.	*Chaetoceros* sp.	*Thalassiosira* sp.
Methanol	**DPPH**	54.41 ± 1.18^a^	46.28 ± 2.60^a^	16.01 ± 0.83^a^	7.62 ± 0.13^a^
	**ABTS**	30.18 ± 1.01^bc^	20.42 ± 1.84^b^	18.74 ± 0.93^a^	11.98 ± 0.57^c^
	**FRAP**	54.64 ± 0.98^e^	60.23 ± 0.43^ab^	47.57 ± 2.43^ab^	27.53 ± 2.78^b^
Ethanol	**DPPH**	40.45 ± 1.36^b^	34.56 ± 1.93^b^	11.42 ± 3.07^b^	5.90 ± 1.88^ab^
	**ABTS**	26.75 ± 1.98^c^	23.07 ± 1.51^b^	11.76 ± 0.84^cd^	9.24 ± 0.51^d^
	**FRAP**	53.64 ± 1.48^e^	65.01 ± 2.54^ab^	40.72 ± 1.10^abc^	26.00 ± 2.28^bc^
Acetone	**DPPH**	34.86 ± 1.44^b^	38.34 ± 1.71^ab^	6.18 ± 0.69^c^	4.06 ± 0.62^bc^
	**ABTS**	32.80 ± 1.00^b^	13.04 ± 1.06^c^	14.54 ± 0.84^bc^	25.07 ± 0.22^b^
	**FRAP**	75.41 ± 1.38^cd^	59.05 ± 3.43^b^	48.52 ± 2.59^a^	42.94 ± 4.32^a^
Chloroform	**DPPH**	36.59 ± 1.02^b^	33.56 ± 1.34^b^	1.08 ± 0.34^d^	0.83 ± 0.04^d^
	**ABTS**	28.00 ± 1.06^bc^	8.92 ± 0.77^cd^	10.01 ± 0.15^d^	8.34 ± 0.33^de^
	**FRAP**	73.25 ± 1.33^d^	20.49 ± 1.48^d^	35.81 ± 1.40^c^	25.88 ± 1.80^bc^
Ethyl acetate	**DPPH**	34.73 ± 1.10^b^	34.88 ± 1.96^b^	5.13 ± 1.21^c^	6.88 ± 4.37^c^
	**ABTS**	41.57 ± 0.83^a^	30.92 ± 1.97^a^	16.67 ± 0.63^ab^	27.97 ± 0.27^a^
	**FRAP**	113.46 ± 4.83^a^	70.68 ± 0.94^a^	36.71 ± 2.92^bc^	41.68 ± 4.30^a^
DCM	**DPPH**	37.83 ± 1.85^b^	39.37 ± 1.97^ab^	1.76 ± 0.54^d^	5.09 ± 1.29^a^
	**ABTS**	26.82 ± 0.92^c^	12.22 ± 1.08^c^	17.37 ± 1.59^ab^	11.46 ± 0.51^c^
	**FRAP**	84.97 ± 2.58^c^	47.12 ± 2.75^c^	37.25 ± 3.54^abc^	35.00 ± 2.27^ab^
Hexane	**DPPH**	2.41 ± 0.28^c^	2.54 ± 0.26^c^	1.02 ± 0.07^d^	ND
	**ABTS**	7.01 ± 0.40^d^	4.43 ± 0.26^d^	8.45 ± 0.15^d^	6.77 ± 0.36^e^
	**FRAP**	96.48 ± 1.39^b^	57.03 ± 2.00^bc^	18.83 ± 1.4^d^	12.74 ± 0.93^c^

*The significant difference among the crude extracts’ DPPH, ABTS and FRAP values in the same column is specified by the superscripts ^(a,b,c,d,e)^.

The ABTS radical scavenging activity in the marine microalgae species was assessed using the linear standard curve of Trolox, (y = 3.0242x + 3.6009; R^2^ = 0.983). Unlike DPPH assay, ethyl acetate extract of all species showed highest ABTS scavenging activity, except *Chaetoceros* sp.. The ethyl acetate extract of *Tetraselmis* sp. showed the highest ABTS scavenging activity (41.57 mg TEAC/ g of extract) in this study, followed by ethyl acetate extract of *Nannochloropsis* sp. (30.92 mg TEAC/ g of extract). Between the diatom species, ethyl acetate extract of *Thalassiosira* sp. showed the highest amount of ABTS scavenging activity (27.97 mg TEAC/ g of extract), while methanol extract of *Chaetoceros* sp. was found to be the highest ABTS scavenger (18.74 mg TEAC/ g of extract). Overall, *Thalassiosira* sp. showed better ABTS scavenging activity than *Chaetoceros* sp. *Nannochloropsis* water extract was the lowest ABTS scavenger (1.59 mg TEAC/ g of extract).

This study also used FRAP assay to determine the ferric-reducing capability of the antioxidants in the marine microalgae species using the linear standard curve of Trolox, (y = 0.0065x + 0.0176; R^2^ = 0.9996). In this study, the ethyl acetate extract of *Tetraselmis* sp. showed the highest ferric-reducing power ((113.46 mg TEAC/ g of extract). Ethyl acetate extract of both green microalgae species showed the highest ferric reduction capacity. But for the diatoms, acetone extract of both species exhibited the highest ferric-reducing capacity. *Chaetoceros* sp. acetone showed better ferric reduction (48.52 mg TEAC/ g of extract) than *Thalassiosira* sp. acetone extract. Hexane extract of *Thalassiosira* sp. showed the lowest ferric reducing power (12.74 mg TEAC/ g of extract) in this assay.

Pearson’s correlation coefficient (r) was acquired by bivariate correlation analysis and the value obtained was used to explain the correlation between antioxidant capability and TPC and TFC. The strength of the correlation was determined by using three coefficient intervals; strong [if r = +/- (0.600–1.000)], moderate [if r = +/- (0.400–0.599)], and weak [if r = +/- (0.000–0.399)].^30^ The correlation estimated between ABTS and TPC was positive and strongly correlated in *Nannochloropsis* sp. (r = 0.777) at p < 0.01 and in the case of TFC, a positive and strong correlation was observed in *Thalassiosira* sp. (r = 0.745) at p < 0.01 and in *Nannochloropsis* sp. (r = 0.619) at p < 0.05. Also, The correlation between FRAP and TFC was positive and strongly correlated in this species (r = 0.762) at p < 0.01.

In *Nannochloropsis* sp., a moderate positive correlation was observed between DPPH vs TFC at (r = 0.567) p < 0.01. Also, a moderate positive correlation was observed between DPPH vs TPC in *Tetraselmis* sp. at (r = 0.487) p < 0.05 and in *Thalassiosira* sp. (r= − 0.487) p < 0.05 (Table 4).

**Table 4:** The linear correlation coefficient among total phenolic (TPC), total flavonoid contents (TFC) and antioxidant assays (DPPH, FRAP, ABTS) of crude extracts of marine green microalgae and diatoms.

Correlation	Correlation coefficient Pearson (*r*)			
	***Tetraselmis* sp.**	***Nannochloropsis* sp.**	***Chaetoceros* sp.**	***Thalassiosira* sp.**
DPPH vs TPC	0.487*	0.367	−0.220	−0.487*
ABTS vs TPC	0.498*	0.777**	0.213	0.157
FRAP vs TPC	−0.330	0.342	0.255	0.095
DPPH vs TFC	0.121	0.567**	0.072	−0.245
ABTS vs TFC	0.335	−0.023	0.354	0.745**
FRAP vs TFC	0.156	0.619*	0.163	0.762**

** significant at *p* < 0.01.

* significant at *p* < 0.05.

### 3.4 Cytotoxicity of marine green microalgae and diatoms crude extracts

**Fig. 1:**
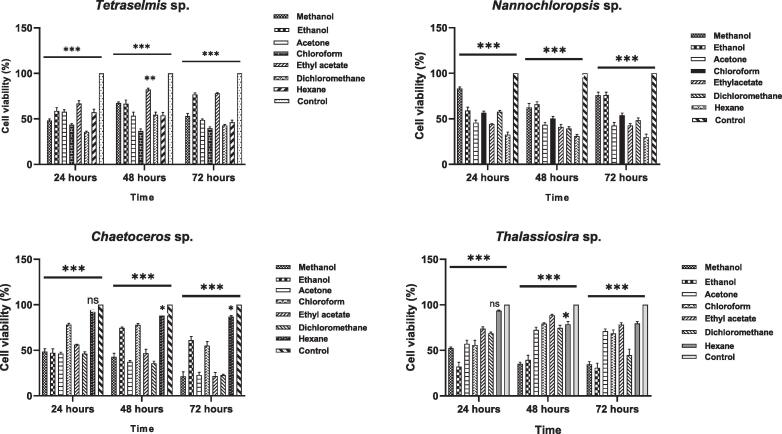
Effects of different solvent extract from green microalgae A. *Tetraselmis* sp., B. *Nannochloropsis* sp., C. *Chaetoceros* sp., D. *Thalassiosira* sp. on the viability of MCF-7 cells with different time points at 100 μg/ml. The means marked with ***, **, * are significantly different at p < 0.0001, p < 0.001 and p < 0.01 compared to the control; ns = not significant.

In this study, seven different polarity solvent extracts with a single concentration (100 µg/ml) were used to test *in vitro* inhibition of the proliferation of MCF-7 cells over different time points. Among different solvent extracts of two green microalgae, *Tetraselmis* sp., and *Nannochloropsis* sp., the hexane extract of *Nannochloropsis* sp. showed higher cytotoxicity against MCF-7 cells (Fig. 1). After 72 h of incubation, this extract reduced the cell viability to 29.80 % with a concentration of 100 μg/ml. Ethyl acetate and acetone extract of *Nannochloropsis* sp. reduced the cell viability to 42.64 % and 42.52 %, respectively at the same concentration and incubation hour. *Tetraselmis* sp. also exhibited cytotoxicity against MCF-7 cells. Chloroform extract of *Tetraselmis* sp. reduced cell viability to 36.52 % after 48 h at a concentration of 100 μg/ml.

In this study, fourteen different solvent extracts of two marine diatom species, *Chaetoceros* sp. and *Thalassiosira* sp., were also evaluated for their cytotoxic activity against the MCF-7 cell line. While comparing these two diatoms, *Chaetoceros* sp. showed more cytotoxicity towards MCF-7 than *Thalassiosira* sp. Methanol and ethyl acetate extract of *Chaetoceros* sp. showed the highest cytotoxicity towards MCF-7. After 72 h of incubation with 100 μg/ml, this diatom reduced cell viability to 21.26 % and 21.56 %, respectively. While acetone and DCM extracts reduced MCF-7 viability to 22.84 % and 22.58 %, respectively. The lowest cytotoxicity was found in the hexane extract where cell viability was reduced only to 86.8 % after 72 h of incubation. On the other hand, ethanol and methanol extracts of *Thalassiosira* sp. reduced cell viability to 30.82 % and 34.69 % with the same concentration and time. Similar to *Chaetoceros* sp., the highest cell viability was recorded with hexane extract of *Thalassiosira* sp. where cell viability was reduced to 79.55 % after 72 h.

## 4 Discussion

Methanol is often considered the most suitable solvent for extracting bioactive metabolites due to its polarity and higher cell disintegrating capacity.^31^ Ethanol is also known as a preferable solvent to extract numerous metabolites of different polarities. On top of that, ethanol is also considered less toxic compared to other solvents. Both of these solvents are known for their high polyphenol extraction efficiency, especially low molecular weight polyphenols and also carotenoids.^32^ Truong et al. (2019) reported that methanol is the best solvent for extracting phenolics, flavonoids, terpenoids and alkaloids compared to other solvents like ethanol, acetone, dichloromethane, and water extract.^33^ In this study, ethanol extract of *Nannochloropsis* sp. showed the highest extraction yield (33 %) which is higher compared to some plant extraction yield using the same solvent.^34^, ^52^, ^53^ This high extraction yield might be attributable to their proper extraction method, time and temperature.^33^, ^35^.

Ethyl acetate extract is also reported to have high polyphenolic content.^36^ Besides polyphenols, polar lipids (digalactosyl diacylglycerols and sulphoquinovosyl diacylglycerols) are found in ethyl acetate.^37^ On the other hand, hexane extract showed low total phenol and flavonoid contents but high carotenoid contents.^38^ Acetone and chloroform extract, on the contrary, have shown good production of polyphenols, carotenoids and fatty acids.^39^ Palaiogiannis et al. (2023) reported that flavonoids were mostly extracted in acetone extract.^40^ Medium polar solvents like ethanol, methanol, acetone, and ethyl acetate extracted more phytochemicals in the current study. Previous studies showed that these less polar solvents extracted more polyphenols compared to non-polar solvents.^31^, ^41^ Besides, polar carotenoids or xanthophylls like lutein, zeaxanthin, violaxanthin, cryptoxanthin, and fucoxanthin can be extracted better in these less polar solvents.^42^ Dichloromethane has shown better extraction of carotenoids like astaxanthin, fucoxanthin, lutein and saturated fatty acids.^43^.

Polyphenolic compounds are attributed to the prime antioxidant defense of plant and algae species, which are also known for their diversified bioactivities with pharmacologic importance, importantly, antioxidant, anticancer, anti-inflammatory, and antimicrobial activity.^44^ These compounds are reported to be found mostly in solvents less polar than water, for instance, ethanol, methanol, acetone, ethyl acetate, or a mixture of these solvents with water.^31^, ^41^ In this study, green microalgae from Chlorophyta, *Tetraselmis* sp. (5.67–19.87 mg GAE/g of extract) showed the highest total phenolic contents. A study by Del Mondo et al. (2021) also recorded that microalgae from Chlorophyta (*Chlorella* sp., *Tetraselmis* sp., *Chlamydomonas* sp., *Scenedesmus* sp., *Dunaliella* sp.) have more phenolics than Ochrophyta (*Nannochloropsis* sp.), which is in line with our findings. They found out that *Tetraselmis* sp. contained TPC of 25.5–0.34 mg GAE/g, which is close to the data of the present study^45^. In the current study, ethanol and ethyl acetate extracts of these two green microalgae showed higher TPC than other solvent extracts. Maadane et al. (2015) also reported TPC in ethanolic extract of *Tetraselmis* sp. (25.5 mg GAE/g of extract). Between the two diatom species, *Chaetoceros* sp. showed more phenolic contents (3.27–15.88 mg GAE/g of extract) than *Thalassiosira* sp. (2.04–4.8 mg GAE/g of extract). Our results for diatom are comparable to the report of Bhattacharjya et al. (2020) where they showed that TPC from *Chaetoceros* sp. is higher than *Thalassiosira* sp.

Flavonoids, a large group of plant polyphenolic metabolites, are incorporated into our diet in large amount. They have a complex molecular structure which can exert several biological functions in the human body. Plant flavonoids are extensively studied for their anticancer activities, along with other biological functions, such as antiaging, antimicrobial, antiradical, UV protection and so on. Microalgae are also an excellent reservoir of flavonoids, though less investigated than phenolic contents.^44^ For extracting TFC, ethanol, methanol, ethyl acetate and acetone are considered favorable solvents, which is confirmed by previous studies.^46^, ^47^, ^48^ Species-wise, diatom *Chaetoceros* sp. and *Thalassiosira* sp. showed lower TFC than green microalgal species, *Tetraselmis* sp. and *Nannochloropsis* sp. in this study. Gnanakani et al. (2019) reported that TFC of *Nannochloropsis* sp. was identified as the highest in ethyl acetate extract (68.77 mg QE/ g), followed by ethanol extract (48.31 mg QE/ g), which is in line with our result. In this study, for *Nannochloropsis* sp., ethyl acetate extract showed the highest TFC (35.04 mg QE/ g of extract) which is followed by ethanol extract (31.48 mg QE/ g of extract). Safafar et al. (2015) measured the TFC in the methanolic extract of *Nannochloropsis salina* which was 3.18 mg QE/g.

For antioxidant activity assays, a concentration of 500 μg/ml was used for all microalgal extracts. The antioxidant capacity of compounds or extracts can be determined using single or multiple concentrations. Since the antioxidative activities of the twenty-eight microalgal extracts were presented in Trolox (standard) equivalent, a single concentration (500 μg/ml) was selected for this study. Several previous studies also used a single concentration of the extracts to present the antioxidative activity of a large number of samples in Trolox equivalent or other standard equivalents.^21^, ^49^, ^50^ DPPH assay is the most commonly used assay to detect the antioxidant activity of an extract or compound.^51^ Overall, green microalgae showed better DPPH scavenging capacity than diatoms in this study. Also, methanol extracts from all species showed better activity compared to others. However, the ethyl acetate extract of *Tetraselmis* showed the highest ABTS radical scavenging activity and ferric reducing capacity which is comparatively higher than some plants.^52^, ^53^ Between the two diatoms, *Chaetoceros* sp. is a good DPPH scavenger compared to *Thalassiosira* sp. Diatoms exhibited less ABTS scavenging capacity than the green microalgae, as well. However, the data obtained from ABTS is aberrant from the DPPH assay. In the ABTS assay, ethyl acetate extracts from these microalgae, except *Chaetoceros* sp., exhibited the highest ABTS scavenging activity. For *Chaetoceros* sp., methanol extract (18.74 mg TEAC/ g of Extract) was the best ABTS scavenger like DPPH. This sensitivity of the ABTS assay may be due to the faster reaction kinetics and high response to the antioxidants.^54^ The highest ferric reduction capacity of ethyl acetate extract has been documented in previous studies as well.^55^, ^56^, ^57^, ^58^ Compounds in ethyl acetate fraction may have a high electron-donating capacity, which is attributable to their high ferric reduction ability and, consequently, good antioxidative properties. However, for both diatoms, *Chaetoceros* sp. and *Thalassiosira* sp., acetone extract exhibited the highest amount of ferric reducing power, 48.52 and 42.94 mg TEAC/ g of extract, respectively. Acetone extracts of diatoms showed high ferric-reducing capacity and free radical scavenging activity in previous studies which might be attributable to their metabolic profile.^39^, ^59^.

The TPC of plants and algae is often considered the main contributor to antioxidant activity. In the current study, the green microalgae showed a moderate correlation between TPC and antioxidant activity (Table 4). Since other phytochemicals like carotenoids, and tocopherols are also responsible for the antioxidant activity of the microalgae, TPC in this study might not be the sole contributor to antioxidant capacity. Besides, the antioxidant activity of an extract may depend on several factors like synergism between antioxidants in the extract, concentration, structure, and interaction between them.^60^ Andriopoulos et al., (2022) argued that pigments like chlorophyll may interfere with the estimation of TPC and antioxidant activity.^61^ In case of TFC, *Nannochloropsis* sp, and *Thalassiosira* sp. showed a strong correlation with antioxidant activity. Previous studies showed that *Nannochloropsis gaditana* contains some flavonoids like catechin, epicatechin 3-O-(4-methylgallate), apigenin-O-rutinoside, 3-methylflavone-8-carboxylic acid, quercetin-3-O-malonylglucoside and rhamnosylhexosyl-methyl-quercetin.^62^, ^63^.

A preliminary *in vitro* cytotoxicity study of the selected marine green microalgae and diatom species was performed against the human breast cancer, MCF-7 cell line. In this study, different solvent extracts with a single concentration (100 µg/ml) were used to test *in vitro* inhibition of proliferation of MCF-7 cells over different time points. Extracts with IC_50_ less than 21 μg/ml are considered as strongly cytotoxic, while IC_50_ between 21 and 200 are considered as moderate cytotoxic.^64^, ^65^ In this study, diatom, *Chaetoceros* sp. showed better cytotoxicity than the green microalgae. The methanol extract of this diatom showed higher cytotoxicity than the rest of the tested extracts. Cytotoxicity of this diatom against breast cancer cells was also documented in previous studies. Goh et al. (2014) reported growth inhibition of MDA-MB-231 by ethyl acetate extract of *C. calcitrans* with IC_50_ of 60 μg/ml after 72 h. Ethanol extract of *C. calcitrans* showed anticancer activity against MCF-7 with IC_50_ of 3 μg/ml after 24 h.^67^ On the contrary, no successful previous report of anticancer activity was found for *Thalassiosira* sp. to date. This study investigated the cytotoxic effect of seven different *Thalassiosira* sp. extracts. Among those extracts, ethanol extract showed the highest cytotoxicity. Hexane extract showed the lowest cytotoxicity for both diatoms.

In case of green microalgae, the hexane extract of *Nannochloropsis* sp. showed higher cytotoxicity. Several reports of the cytotoxic potential of *Nannochloropsis* spp. against breast cancer cells have been made where different extracts or partially purified products showed anti-cancer activity. Fatty acid potassium salts (FAPS), derived from *N. salina* showed marked suppression (IC_50_ = 0.45 µg/mL) on MCF-7 cells in a dose-dependent manner, which was attributed to the presence of dihomo-γ-linolenic acid (DGLA) and eicosapentaenoic (EPA) acids in FAPS.^68^ Wali et al. (2020) reported the cytotoxic potential of the methanol extract of *N. oculata.* At 200 μg/ml, cell viability of MDA-MB-231 breast cancer cells reduced to 25 % after 72 h^69^. Methanol extract of *N. oceanica* extract exhibited cytotoxicity of 46.86 % against MCF-7 cells at 200 μg/ml.^70^ *Nannochloropsis* spp. contain different phytochemicals which are attributable to their cytotoxicity. Kim et al., (2021) reported the presence of fatty acids and carotenoids like violaxanthin, astaxanthin, zeaxanthin, canthaxanthin, and β-carotene which are known for their bioactivities like antioxidative and anticancer properties.^71^, ^72^ Among the tested extracts of *Tetraselmis* sp., chloroform extract showed the best cytotoxicity. Our data is in agreement with the previous study by.^73^ They reported that chloroform extract of *T. suecica* inhibited MCF-7 cells with IC_50_ of 46.77 μg/ml after 72 h. *Tetraselmis* species may contain different pigments and fatty acids. *T. chuii* was reported to contain chlorophyll *b*, lutein, EPA and linolenic acid.^74^ However, microalgae tested in this study have been reported to show less toxicity towards non-cancerous cell lines in previous studies.^75^, ^73^, ^76^, ^77^, ^66^.

Azizan et al., 2020 reported the presence of ten carotenoids in *C. calcitrans,* thirteen fatty acids including EPA and DHA, and sixteen lipids including glycerolipids, glycerolphospholipids and sterol. Most of these carotenoids have previous cytotoxicity reports.^78^ For example, fucoxanthin showed an anticancer effect against different cell lines. Ahmed et al., 2023 reported the anticancer effect of fucoxanthin (fx) in triple-negative breast cancer cells, MDA-MB-231 and MDA-MB-468 where fx induced apoptosis in cancer cells and also inhibited angiogenesis.^79^ Fatty acids like EPA and DHA also showed anti-breast cancer activity through apoptosis and were reported to inhibit angiogenesis ^80^(Brown et al., 2020). Moreover, polar solvents like methanol contain more phenolics which are another contributor to the cytotoxic effect in cancer cells.^81^ Therefore, the presence of these metabolites may play a significant role in exerting anti-breast cancer activity of *Chaetoceros* sp.

As discussed earlier, microalgae contain several classes of phytochemicals, like carotenoids, fatty acids, phenolic and flavonoids which may attributed to their cytotoxic properties. Studies showed that mixtures of these phytochemicals acted better on cancer cells because of having synergistic and additive effects, structural stabilizing effects, and high bioavailability effects which contributed to high therapeutic efficiency by targeting different pathways.^82^, ^83^ The mechanism of cytotoxicity of these microalgae extracts may involve scavenging free radicals upon entering the cancer cells and thus alter the antioxidant status which leads to activation of signaling molecules of different pathways. This activation of cellular proteins may regulate cellular defence mechanisms which help inhibit cell proliferation and inducing apoptosis. They can also inactivate carcinogens.^84^, ^85^, ^86^ However, some studies argued that free radical scavenging activity may not be correlated with the cytotoxicity of plant extract.^87^, ^88^, ^89^ In this study, the green microalgae showed better free radical scavenging activity but less cytotoxicity than the diatom, *Chaetoceros* sp. Therefore, the cytotoxicity mechanism along with the exact cytotoxic compounds needs to be investigated in future studies.

## 5 Conclusion

The global market for marine-based therapeutics is burgeoning which makes more demand to explore largely uninvestigated marine eukaryotic microalgae to discover new and more potential bioactive metabolites. This study explored bioactivities of eukaryotic microalgae from marine origin to minimize the knowledge gap and also a comparison between green microalgae and diatoms is given for a better understanding of their bioactive properties. The current study suggested that green microalga, *Tetraselmis* sp., and diatom, *Chaetoceros* sp. have the most potential in terms of antioxidant and cytotoxic activities, respectively. Among the seven solvents tested, medium polar solvents like methanol, and ethyl acetate were recommended for efficient extraction of bioactive compounds. The antioxidant and cytotoxic activity were found to be higher in these solvent extracts. Analysis of the total phenolic and flavonoid contents of the microalgae also suggested that ethyl acetate is the preferred solvent for extracting polyphenolic compounds. Besides, green microalgae showed better antioxidative capabilities compared to diatoms. On the contrary, diatom, *Chaetoceros* sp. showed better cytotoxicity than the green microalgae. Moreover, the cytotoxic effect of *Thalassiosira* sp. against breast cancer cells was also documented in this study. It is recommended to investigate the metabolic profile of these microalgae and evaluate *in vivo* antioxidant and cytotoxic activities. Also, the apoptosis mechanism of *Chaetoceros* sp. needs to be investigated to better understand the targets of cellular death.

## CRediT authorship contribution statement

**Umme Tamanna Ferdous:** Writing – review & editing, Writing – original draft, Validation, Methodology, Investigation, Formal analysis, Data curation, Conceptualization. **Armania Nurdin:** Writing – review & editing, Validation, Methodology, Conceptualization. **Saila Ismail:** Writing – review & editing, Validation, Conceptualization. **Khozirah Shaari:** Writing – review & editing, Validation, Conceptualization. **Zetty Norhana Balia Yusof:** Writing – review & editing, Validation, Supervision, Project administration, Funding acquisition, Conceptualization.

## Declaration of competing interest

The authors declare that they have no known competing financial interests or personal relationships that could have appeared to influence the work reported in this paper.

## Data Availability

The datasets analyzed during the current study are available from the corresponding author on reasonable request.

## References

[1] Wang E., Sorolla M.A., Krishnan P.D.G., Sorolla A. (2020). From seabed to bedside: a review on promising marine anticancer compounds. Biomolecules.

[2] Khalifa S.A.M., Elias N., Farag M.A. (2019). Marine natural products: a source of novel anticancer drugs. Mar Drugs.

[3] Sithranga Boopathy N., Kathiresan K. (2010). Anticancer drugs from marine flora: an overview. J Oncol.

[4] Gong M., Bassi A. (2016). Carotenoids from microalgae: a review of recent developments. Biotechnol Adv.

[5] Rajkumar R., Yaakob Z., Takriff M.S. (2014). Potential of the micro and macro algae for biofuel production: a brief review. BioResources.

[6] Li Y., Wang C., Liu H. (2020). Production, isolation and bioactive estimation of extracellular polysaccharides of green microalga Neochloris oleoabundans. Algal Res.

[7] Sansone C., Brunet C. (2019). Promises and challenges of microalgal antioxidant production. Antioxidants.

[8] Abd El-Hack M.E., Abdelnour S., Alagawany M. (2019). Microalgae in modern cancer therapy: current knowledge. Biomed Pharmacother.

[9] Martínez Andrade K.A., Lauritano C., Romano G., Ianora A. (2018). Marine microalgae with anti-cancer properties. Mar Drugs.

[10] Hamidi M., Safarzadeh Kozani P., Safarzadeh Kozani P., Pierre G., Michaud P., Delattre C. (2020). Marine bacteria versus microalgae: Who is the best for biotechnological production of bioactive compounds with antioxidant properties and other biological applications?. Mar Drugs.

[11] Lucakova S., Branyikova I., Hayes M. (2022). Microalgal proteins and bioactives for food, feed, and other applications. Appl Sci.

[12] Yasir S., Siddiki A., Mofijur M. (2022). Microalgae biomass as a sustainable source for biofuel , biochemical and biobased value-added products : an integrated biorefinery concept. Fuel.

[13] Camacho F., Macedo A., Malcata F. (2019). Potential industrial applications and commercialization of microalgae in the functional food and feed industries: a short review. Mar Drugs.

[14] Lafarga T. (2019). Effect of microalgal biomass incorporation into foods : nutritional and sensorial attributes of the end products. Algal Res.

[15] Nethravathy M.U., Mehar J.G., Mudliar S.N., Shekh A.Y. (2019). Recent advances in microalgal bioactives for food, feed, and healthcare products: commercial potential, market space, and sustainability. Compr Rev Food Sci Food Saf.

[16] Calvani M., Pasha A., Favre C. (2020). Nutraceutical boom in cancer : inside the labyrinth of reactive oxygen species. Int J Mol Sci.

[17] Natrah F.M.I., Yusoff F.M., Shariff M., Abas F., Mariana N.S. (2007). Screening of Malaysian indigenous microalgae for antioxidant properties and nutritional value. J Appl Phycol.

[18] Bustamam M.S.A., Pantami H.A., Azizan A. (2021). Complementary analytical platforms of NMR spectroscopy and LCMS analysis in the metabolite profiling of isochrysis galbana. Mar Drugs.

[19] Azizan A., Maulidiani M., Rudiyanto R. (2020). Mass spectrometry-based metabolomics combined with quantitative analysis of the microalgal diatom (Chaetoceros calcitrans). Mar Drugs.

[20] Farahin A.W., Yusoff F.M., Nagao N., Basri M., Shariff M. (2016). Phenolic content and antioxidant activity of Tetraselmis tetrahale (West) Butcher 1959 cultured in annular photobioreactor. J Environ Biol.

[21] Foo S.C., Yusoff F.M., Ismail M. (2015). Efficient solvent extraction of antioxidant-rich extract from a tropical diatom, Chaetoceros calcitrans (Paulsen) Takano 1968. Asian Pac J Trop Biomed.

[22] Goh S.-H., Yusoff F.M., Loh S.P. (2010). A comparison of the antioxidant properties and total phenolic content in a diatom, chaetoceros sp. and a green microalga, nannochloropsis sp. J Agric Sci.

[23] Sansone C., Galasso C., Orefice I. (2017). The green microalga Tetraselmis suecica reduces oxidative stress and induces repairing mechanisms in human cells. Sci Rep.

[24] Moreau D., Tomasoni C., Jacquot C. (2006). Cultivated microalgae and the carotenoid fucoxanthin from Odontella aurita as potent anti-proliferative agents in bronchopulmonary and epithelial cell lines. Environ Toxicol Pharmacol.

[25] Trung T.S., Huyen N.T.K., Minh N.C., Le Trang T.T., Han N.T. (2016). Optimization of harvesting of microalgal Thalassiosira pseudonana biomass using chitosan prepared from shrimp shell waste. Asian J Agric Res.

[26] Hussein H.A., Abdullah M.A. (2020). Anticancer compounds derived from marine diatoms. Mar Drugs.

[27] Bhattacharjya R., Kiran Marella T., Tiwari A., Saxena A., Kumar Singh P., Mishra B. (2020). Bioprospecting of marine diatoms Thalassiosira, Skeletonema and Chaetoceros for lipids and other value-added products. Bioresour Technol.

[28] Hossain N., Hasan M.H., Mahlia T.M.I., Shamsuddin A.H., Silitonga A.S. (2020). Feasibility of microalgae as feedstock for alternative fuel in Malaysia: a review. Energy Strateg Rev.

[29] Ferdous U.T., Nurdin A., Ismail S., Yusof Z.N.B. (2022). Evaluation of the antioxidant and cytotoxic activities of crude extracts from marine Chlorella sp. Biocatal Agric Biotechnol.

[30] Ngamdee P., Wichai U., Jiamyangyuen S. (2016). Correlation between phytochemical and mineral contents and antioxidant activity of black glutinous rice bran, and its potential chemopreventive property. Food Technol. Biotechnol.

[31] Safafar H., Van W.J., Møller P., Jacobsen C. (2015). Carotenoids, phenolic compounds and tocopherols contribute to the antioxidative properties of some microalgae species grown on industrial wastewater. Mar Drugs.

[32] Maadane A., Merghoub N., Ainane T. (2015). Antioxidant activity of some Moroccan marine microalgae: Pufa profiles, carotenoids and phenolic content. J Biotechnol.

[33] Truong D.H., Nguyen D.H., Ta N.T.A., Bui A.V., Do T.H., Nguyen H.C. (2019). Evaluation of the use of different solvents for phytochemical constituents, antioxidants, and in vitro anti-inflammatory activities of Severinia buxifolia. J Food Qual.

[34] Kurt-Celep İ., Zengin G., Uba A.I. (2023). Unraveling the chemical profile, antioxidant, enzyme inhibitory, cytotoxic potential of different extracts from Astragalus caraganae. Arch Pharm (Weinheim).

[35] Rokicka M., Zieliński M., Dudek M., Dębowski M. (2021). Effects of ultrasonic and microwave pretreatment on lipid extraction of microalgae and methane production from the residual extracted biomass. Bioenergy Res.

[36] Goiris K., Muylaert K., Fraeye I., Foubert I., De Brabanter J., De Cooman L. (2012). Antioxidant potential of microalgae in relation to their phenolic and carotenoid content. J Appl Phycol.

[37] Iglesias M.J., Soengas R., Probert I., Guilloud E., Gourvil P., Mehiri M. (2019). NMR characterization and evaluation of antibacterial and antiobiofilm activity of organic extracts from stationary phase batch cultures of five marine microalgae (Dunaliella sp., D. salina, Chaetoceros calcitrans, C. gracilis and Tisochrysis lutea). Phytochemistry.

[38] Thavamoney N., Sivanadian L., Tee L.H., Khoo H.E., Prasad K.N., Kong K.W. (2018). Extraction and recovery of phytochemical components and antioxidative properties in fruit parts of Dacryodes rostrata influenced by different solvents. J Food Sci Technol.

[39] Azizan A., Bustamam M.S.A., Maulidiani M. (2018). Metabolite profiling of the microalgal diatom chaetoceros calcitrans and correlation with antioxidant and nitric oxide inhibitory Activities via 1H NMR-Based Metabolomics. Mar Drugs.

[40] Palaiogiannis D., Chatzimitakos T., Athanasiadis V., Bozinou E., Makris D.P., Lalas S.I. (2023). Successive solvent extraction of polyphenols and flavonoids from cistus creticus L. Leaves. Oxygen.

[41] Ferreira R.M., Ribeiro A.R., Patinha C., Silva A.M.S., Cardoso S.M., Costa R. (2019). Water extraction kinetics of bioactive compounds of fucus vesiculosus. Molecules.

[42] Kultys E., Kurek M.A. (2022). Green extraction of carotenoids from fruit and vegetable byproducts: a review. Molecules.

[43] Pan Y.L., Rodrigues M.J., Pereira C.G. (2021). Exploring the biotechnological value of marine invertebrates: A closer look at the biochemical and antioxidant properties of sabella spallanzanii and microcosmus squamiger. Animals.

[44] Ferdous U.T., Yusof Z.N.B. (2021). Insight into potential anticancer activity of algal flavonoids : current status and challenges. Molecules.

[45] Del Mondo A., Smerilli A., Ambrosino L. (2021). Insights into phenolic compounds from microalgae: structural variety and complex beneficial activities from health to nutraceutics. Crit Rev Biotechnol.

[46] Nguyen N.V.T., Duong N.T., Nguyen K.N.H. (2022). Effect of extraction solvent on total phenol, flavonoid content, and antioxidant activity of avicennia officinalis. Biointerface Res Appl Chem.

[47] Tan L.T. (2013). Marine cyanobacteria: a prolific source of bioactive natural products as drug leads. Mar Microbiol Bioact Compd Biotechnol Appl.

[48] Gnanakani P.E., Santhanam P., Kumar K.E., Dhanaraju M.D. (2019). Chemical composition, antioxidant, and cytotoxic potential of nannochloropsis species extracts. J Nat Sci Biol Med.

[49] Coronado-Reyes J.A., Acosta-Ramírez E., Martínez-Olguín M.V., González-Hernández J.C. (2022). Antioxidant activity and kinetic characterization of chlorella vulgaris growth under flask-level photoheterotrophic growth conditions. Molecules.

[50] Gauthier M.R., Senhorinho G.N.A., Basiliko N., Desjardins S., Scott J.A. (2022). Green photosynthetic microalgae from low pH environments associated with mining as a potential source of antioxidants. Ind Biotechnol.

[51] Sadeer N.B., Montesano D., Albrizio S., Zengin G., Mahomoodally M.F. (2020). The versatility of antioxidant assays in food science and safety—chemistry, applications, strengths, and limitations. Antioxidants.

[52] Acquaviva A., Nilofar B.A., Zengin G., Di Simone S.C., Recinella L. (2023). Chemical characterization of different extracts from artemisia annua and their antioxidant, enzyme inhibitory and anti-inflammatory properties. Chem Biodivers.

[53] Zengin G., Fernández-Ochoa Á., de la Luz C.-G. (2024). Cytotoxic, antioxidant, and enzyme inhibitory activities of Centaurea stapfiana extracts and their HPLC-ESI-QTOF-MS profiles: Insights into an unexplored Centaurea species. Fitoterapia.

[54] Lee K.J., Oh Y.C., Cho W.K., Ma J.Y. (2015). Antioxidant and anti-inflammatory activity determination of one hundred kinds of pure chemical compounds using offline and online screening HPLC assay. Evidence-Based Complement Altern Med.

[55] Assefa A.D., Ko E.Y., Moon S.H., Keum Y.S. (2016). Antioxidant and antiplatelet activities of flavonoid-rich fractions of three citrus fruits from Korea. 3 Biotech.

[56] Wan Rosli W.I., Siti Nur Haffizah R., Nurraihana H. (2018). Ant oxidative and scavenging properties of polyphenolic rich-fraction of cornett’s (Young zea mays). Int J Recent Technol Eng.

[57] Park M., Kim M. (2017). Analysis of antioxidant and anti-inflammatory activities of solvent fractions from rhynchosia nulubilis cultivated with ganoderma lucidum mycelium. Prev Nutr Food Sci.

[58] Ismail A., Prasad K.N., Chew L.Y., Khoo H.E., Kong K.W., Azlan A. (2010). Antioxidant capacities of peel, pulp, and seed fractions of canarium odontophyllum Miq. fruit. J Biomed Biotechnol.

[59] Karthikeyan P. (2013). In vitro antioxidant activity of marine diatoms. IOSR J Environ Sci Toxicol Food Technol.

[60] Djordjevic T.M., Šiler-Marinkovic S.S., Dimitrijevic-Brankovic S.I. (2011). Antioxidant activity and total phenolic content in some cereals and legumes. Int J Food Prop.

[61] Andriopoulos V., Gkioni M.D., Koutra E. (2022). Total phenolic content , biomass composition , and antioxidant activity of selected marine microalgal species with potential as aquaculture feed. Antioxidants.

[62] Martínez R., García-Beltrán A., Kapravelou G. (2022). In vivo nutritional assessment of the microalga nannochloropsis gaditana and evaluation of the antioxidant and antiproliferative capacity of its functional extracts. Mar Drugs.

[63] Haoujar I., Cacciola F., Abrini J. (2019). Mediterranean Morocco.

[64] Widiandani T., Tandian T., Zufar B.D. (2023). In vitro study of pinostrobin propionate and pinostrobin butyrate: cytotoxic activity against breast cancer cell T47D and its selectivity index. J Public Health Africa.

[65] Zulkipli N.N., Rahman S.A., Taib W.R.W. (2024). The cytotoxicity effect and identification of bioactive compounds of Prismatomeris glabra crude leaf extracts against breast cancer cells. Beni-Suef Univ J Basic Appl Sci.

[66] Goh S., Alitheen N.B., Yusoff F., Yap S., Loh S. (2014). Crude ethyl acetate extract of marine microalga, Chaetoceros calcitrans, induces Apoptosis in MDA-MB-231 breast cancer cells. Pharmacogn Mag.

[67] Ebrahimi Nigjeh S., Yusoff F.M., Mohamed Alitheen N.B., Rasoli M., Keong Y.S., Bin O.AR. (2013). Cytotoxic effect of ethanol extract of microalga, Chaetoceros calcitrans, and its mechanisms in inducing apoptosis in human breast cancer cell line. Biomed Res Int.

[68] Sayegh F., Elazzazy A., Bellou S. (2016). Production of polyunsaturated single cell oils possessing antimicrobial and anticancer properties. Ann Microbiol.

[69] Wali A.F., Al D.Y., Pillai J.R. (2020). Lc-ms phytochemical screening, in vitro antioxidant, antimicrobial and anticancer activity of microalgae nannochloropsis oculata extract. Separations.

[70] Elkhateeb W., El-Sayed H., Fayad W., Al K.AG., Emam M., Daba G. (2020). In vitro Anti-breast cancer and antifungal Bio-efficiency of some microalgal extracts Waill. J Membr Sci Res.

[71] Kim S.Y., Kwon Y.M., Kim K.W., Young J., Kim H. (2021). Exploring the Potential of Nannochloropsis sp. Extract for Cosmeceutical Applications. Mar Drugs.

[72] Ferdous U.T., Yusof Z.N.B. (2021). Medicinal prospects of antioxidants from algal sources in cancer therapy. Front Pharmacol.

[73] Hussein H.A., Mohamad H., Ghazaly M.M., Laith A.A., Abdullah M.A. (2019). Cytotoxic effects of Tetraselmis suecica chloroform extracts with silver nanoparticle co-application on MCF-7, 4 T1, and Vero cell lines. J Appl Phycol.

[74] Herbert H., Parkes R., Barone M.E. (2024). Antioxidant properties and bioactivity of three marine microalgae on human cancer cell lines. Appl Phycol.

[75] Custódio L., Soares F., Pereira H. (2014). Fatty acid composition and biological activities of Isochrysis galbana T-ISO, Tetraselmis sp. and Scenedesmus sp.: Possible application in the pharmaceutical and functional food industries. J Appl Phycol.

[76] Sanjeewa K.K.A., Fernando I.P.S., Samarakoon K.W. (2016). Anti-inflammatory and anti-cancer activities of sterol rich fraction of cultured marine microalga nannochloropsis oculata. Algae.

[77] Foo S.C., Yusoff F.M., Imam M.U. (2018). Increased fucoxanthin in Chaetoceros calcitrans extract exacerbates apoptosis in liver cancer cells via multiple targeted cellular pathways. Biotechnol Reports.

[78] Ferdous U.T., Yusof Z.N.B. (2021). Algal terpenoids: a potential source of antioxidants for cancer therapy. Terpenes and Terpenoids, IntechOpen.

[79] Ahmed S.A., Mendonca P., Messeha S.S., Soliman K.F.A. (2023). Anticancer effects of fucoxanthin through cell cycle arrest, apoptosis induction, and angiogenesis inhibition in triple-negative breast cancer cells. Molecules.

[80] Brown I., Lee J., Sneddon A.A. (2020). Anticancer effects of n-3 EPA and DHA and their endocannabinoid derivatives on breast cancer cell growth and invasion. Prostaglandins Leukot Essent Fat Acids.

[81] Bakrim S., El Omari N., El Hachlafi N., Bakri Y., Lee L.H., Bouyahya A. (2022). Dietary phenolic compounds as anticancer natural drugs: recent update on molecular mechanisms and clinical trials. Foods.

[82] Aung T.N., Qu Z., Kortschak R.D., Adelson D.L. (2017). Understanding the effectiveness of natural compound mixtures in cancer through their molecular mode of action. Int J Mol Sci.

[83] Kapinova A., Stefanicka P., Kubatka P. (2017). Are plant-based functional foods better choice against cancer than single phytochemicals? a critical review of current breast cancer research. Biomed Pharmacother.

[84] George B.P., Chandran R., Abrahamse H. (2021). Role of phytochemicals in cancer chemoprevention: insights. Antioxidants.

[85] Choudhari A.S., Mandave P.C., Deshpande M., Ranjekar P., Prakash O. (2020). Phytochemicals in cancer treatment: From preclinical studies to clinical practice. Front Pharmacol.

[86] Meybodi N.M., Mortazavian A.M., Monfared A.B., Sohrabvandi S., Meybodi F.A. (2017). Phytochemicals in cancer prevention: a review of the evidence. Int J Cancer Manag.

[87] Sammar M., Abu-Farich B., Rayan I., Falah M., Rayan A. (2019). Correlation between cytotoxicity in cancer cells and free radical-scavenging activity: In vitro evaluation of 57 medicinal and edible plant extracts. Oncol Lett.

[88] Milella R.A., De Rosso M., Gasparro M. (2023). Correlation between antioxidant and anticancer activity and phenolic profile of new Apulian table grape genotypes (V. Vinifera L.). Front. Plant Sci.

[89] Grigalius I., Petrikaite V. (2017). Relationship between antioxidant and anticancer activity of trihydroxyflavones. Molecules.

