# Supplementary material for: A comparative study on antioxidant properties, total phenolics, total flavonoid contents, and cytotoxic properties of marine green microalgae and diatoms
Source: J Genet Eng Biotechnol. 2025 Jan 22;23(1):100456. doi: 10.1016/j.jgeb.2024.100456 (PMC11795137; doi:10.1016/j.jgeb.2024.100456)
Supplement: Supplementary Data 1 [file mmc1.docx]

**Supplementary materials**

**Supplementary Table 1**

**ST 1.** Composition of F/2 growth media

| Stock Solution | Ingredients | Amount (g) | 1L medium (mL) | |
| --- | --- | --- | --- | --- |
| Solution A | NaNO_3_  Distilled water (mL) | 75  1000 | | 1 |
| Solution B | NaH_2_PO_4_.H_2_O  Distilled Water (mL) | 5  1000 | | 1 |
| *Solution C | \| Na_2_SiO_3_・9H2O \| \| --- \| \| Distilled Water (mL) \| | \| 15 \| \| --- \| \| 500 \| | | 1 |
| Solution D | \| FeCl_3_.6H_2_O \| \| --- \| \| Na_2_EDTA.2H_2_O \| \| MnCl_2_.4H_2_O \| \| ZnSO_4_.7H_2_O \| \| CoCl_2_.6H_2_O \| \| CuSO_4_.5H_2_O \| \| Na_2_MoO_4_.2H_2_O \| \| Distilled Water (mL) \| | \| 3.15 \| \| --- \| \| 4.36 \| \| 0.18 \| \| 0.22 \| \| 0.01 \| \| 0.098 \| \| 0.063 \| \| 1000 \| | | 1 |
| Solution E | \| Thiamine.HCl \| \| \| --- \| --- \| \| Biotin \|  \| \| Cyanocobalamin  Distilled Water (mL) \| \| | \| 0.2 \| \| --- \| \| 0.001 \| \| 0.001  1000 \| | | 0.5 |

*Solution C is only for Diatoms (*Chaetoceros, Thalassiosira*)

**Supplementary Figure 1**

| **A**A 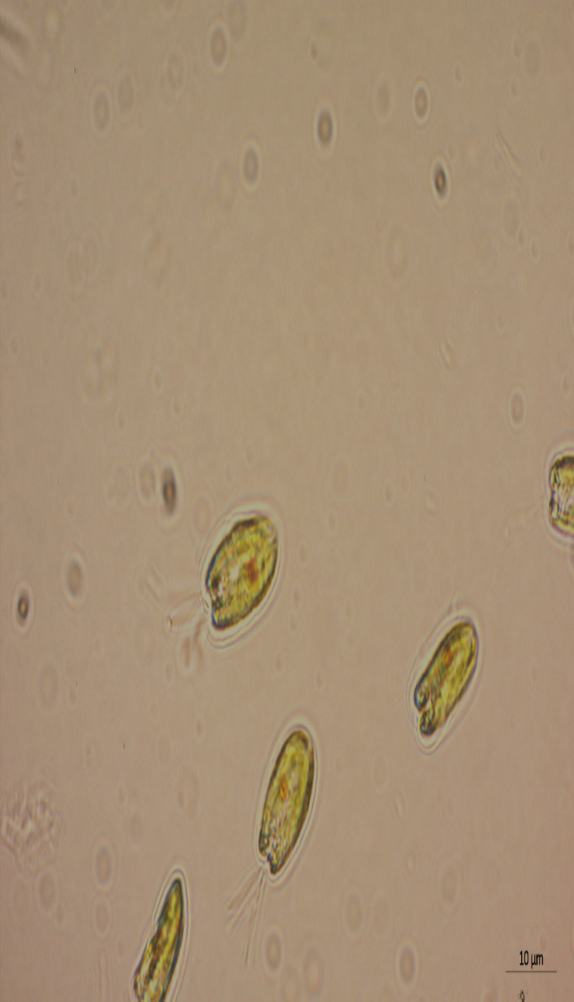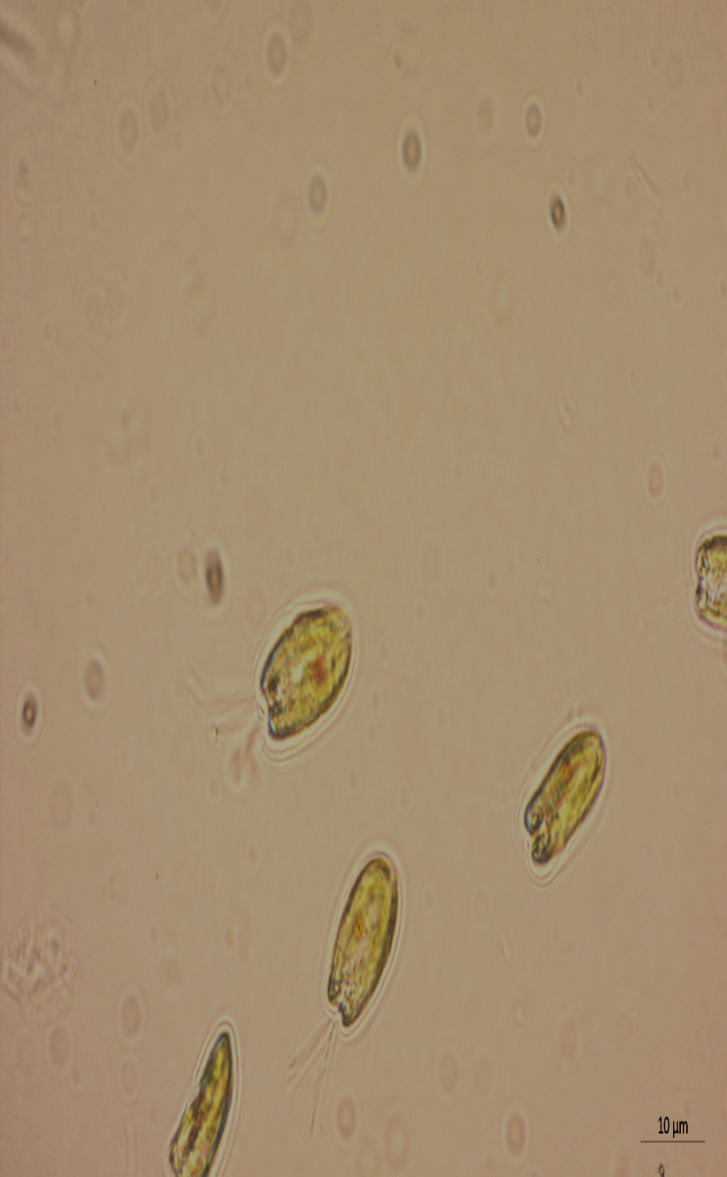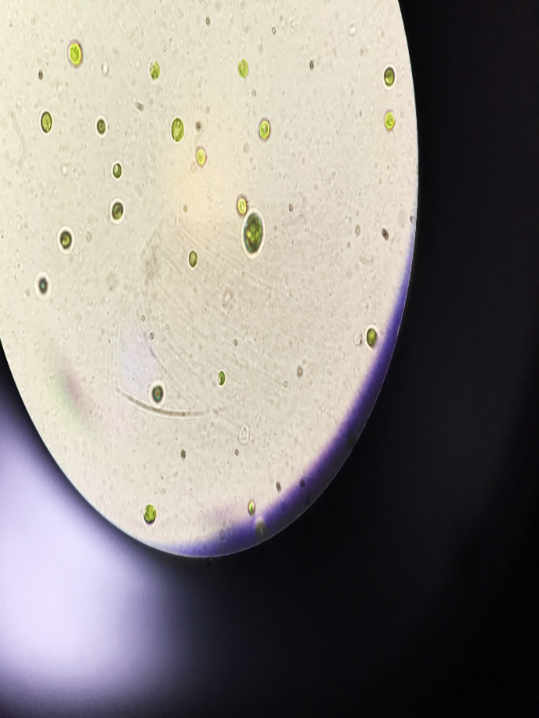 | **B**A 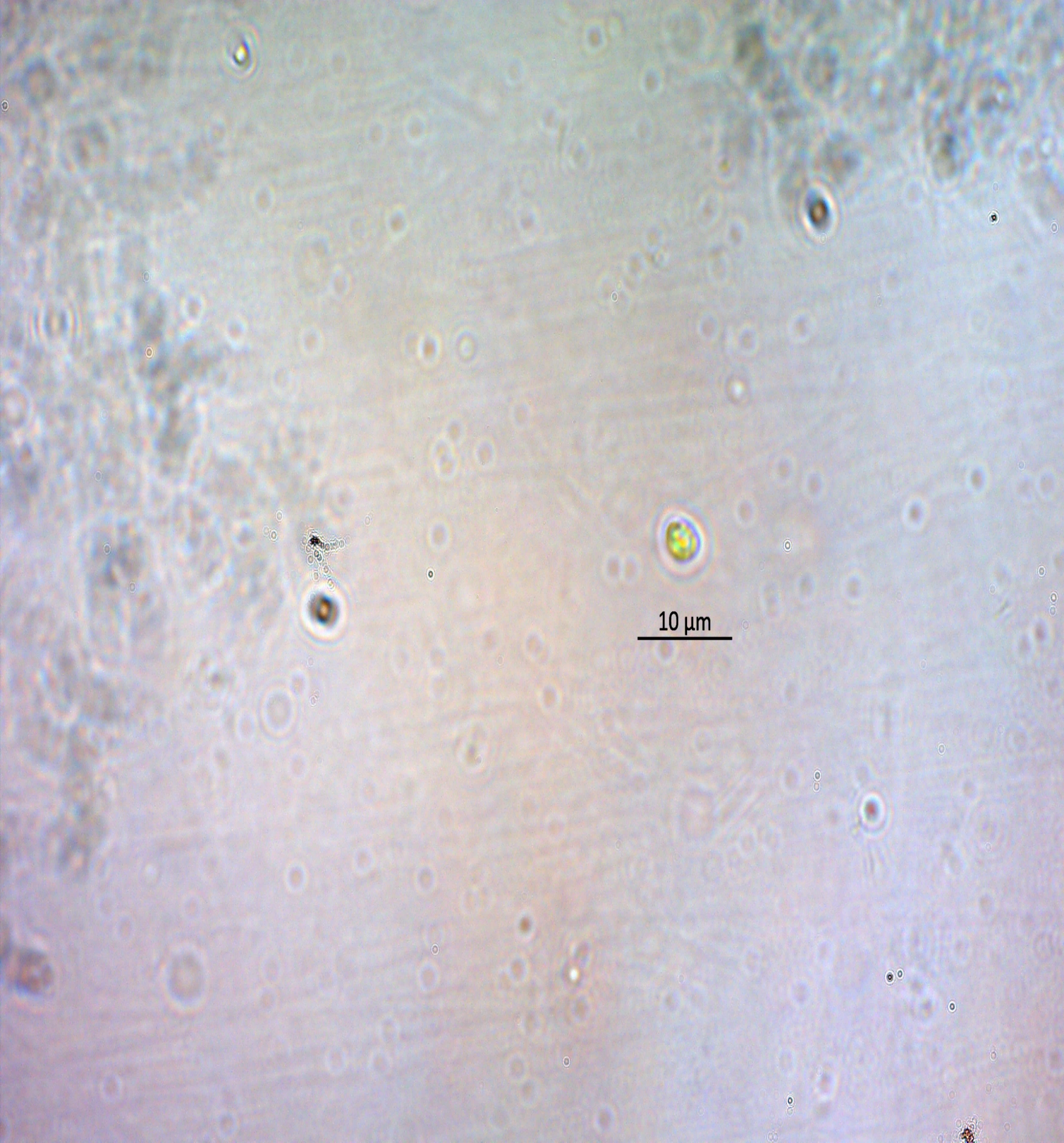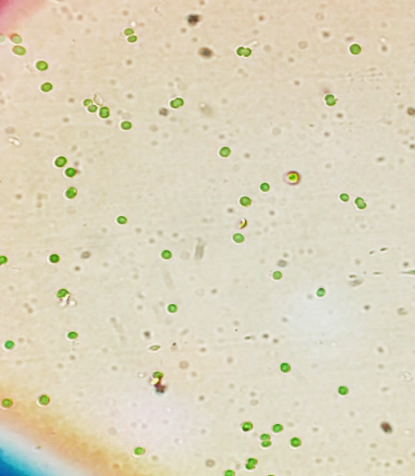 |
| --- | --- |
| **C**A  **B**A 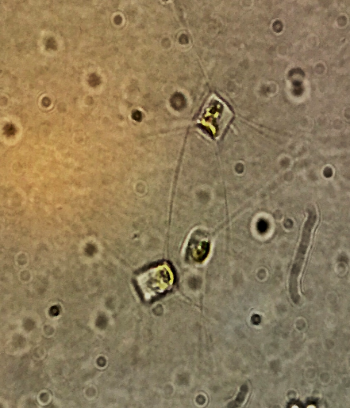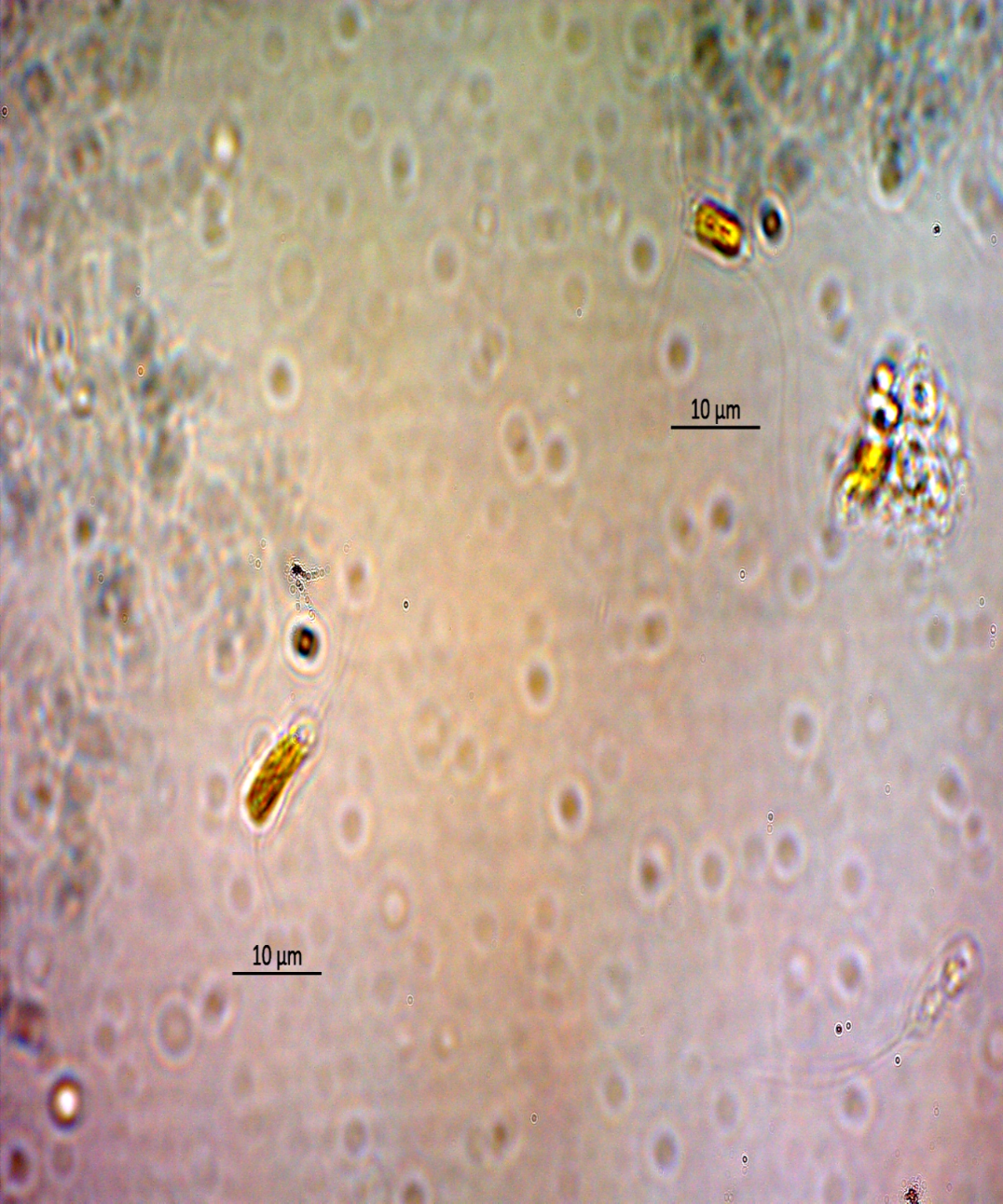 | **D**A 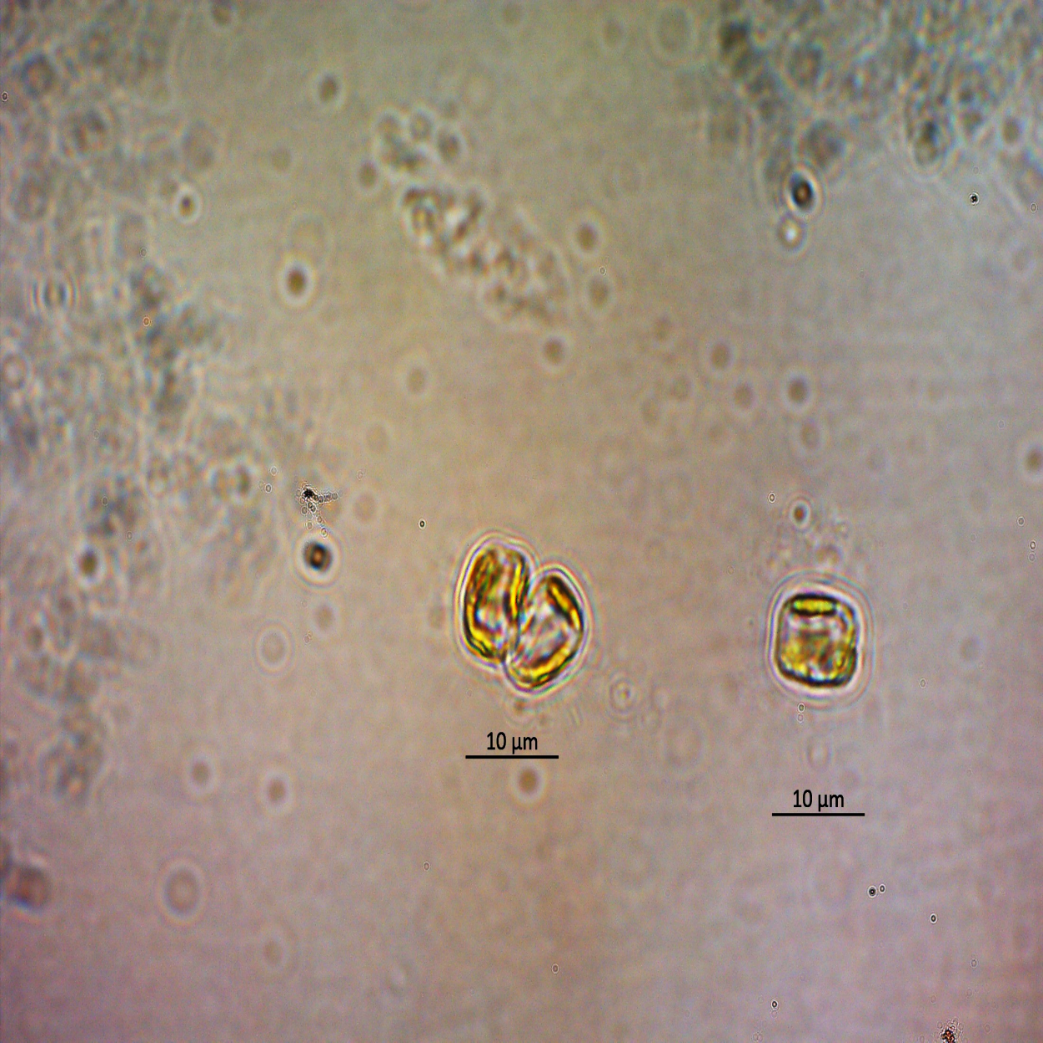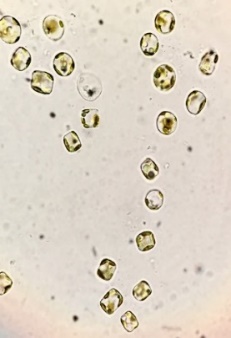 |

**SF 1**. Micrograph of (A) *Tetraselmis* sp.; (B) *Nannochloropsis* sp.; (C) *Chaetoceros* sp.; (D) *Thalassiosira* sp.; micrographs on left side are of 100x and right side are of 1000x magnification
